# What Role Can Avatars Play in e-Mental Health Interventions? Exploring New Models of Client–Therapist Interaction

**DOI:** 10.3389/fpsyt.2016.00186

**Published:** 2016-11-18

**Authors:** Imogen C. Rehm, Emily Foenander, Klaire Wallace, Jo-Anne M. Abbott, Michael Kyrios, Neil Thomas

**Affiliations:** ^1^National eTherapy Centre, Centre for Mental Health, Swinburne University of Technology, Melbourne, VIC, Australia; ^2^Research School of Psychology, College of Medicine, Biology and Environment, Australian National University, Canberra, ACT, Australia; ^3^Monash Alfred Psychiatry Research Centre, Monash University Central Clinical School and The Alfred, Melbourne, VIC, Australia

**Keywords:** avatars, virtual environments, virtual reality, e-mental health, digital mental health, human–computer interaction, computer-mediated communication

## Abstract

In the burgeoning field of e-mental health interventions, avatars are increasingly being utilized to facilitate online communication between clients and therapists, and among peers. Avatars are digital self-representations, which enable individuals to interact with each other in computer-based virtual environments. In this narrative review, we examine the psychotherapeutic applications of avatars that have been investigated and trialed to date. Five key applications were identified (1) in the formation of online peer support communities; (2) replicating traditional modes of psychotherapy by using avatars as a vehicle to communicate within a wholly virtual environment; (3) using avatar technology to facilitate or augment face-to-face treatment; (4) as part of serious games; and (5) communication with an autonomous virtual therapist. Across these applications, avatars appeared to serve several functions conducive to treatment engagement by (1) facilitating the development of a virtual therapeutic alliance; (2) reducing communication barriers; (3) promoting treatment-seeking through anonymity; (4) promoting expression and exploration of client identity; and (5) enabling therapists to control and manipulate treatment stimuli. Further research into the feasibility and ethical implementation of avatar-based psychotherapies is required.

## Introduction

In online virtual environments, multiple remotely located users can synchronously communicate and interact with each other *via* an avatar – a digital character that the user can customize to represent his/her identity. In their seminal 2008 paper, Gorini et al. (1) proposed two key applications of avatars in multiuser, computer-based, three-dimensional virtual worlds, such as *Second Life*, which could potentially transform the delivery of online interventions for mental health problems (i.e., e-mental health interventions). These included (1) an alternative form of technology (i.e., other than head-mounted virtual reality devices) to deliver exposure-based therapy for anxiety disorders and substance-abuse problems and (2) facilitation of online peer support communities. Studies since 2008 have proposed several further applications of avatars in the delivery of e-mental health interventions. For instance, head-mounted virtual reality devices that immerse clients into computer-generated interactions with avatars are increasingly being utilized to treat anxiety disorders (2) and persecutory delusions (3). Indeed, immersive virtual reality technology has previously been reviewed for its significant potential in psychiatric applications (4, 5). In this narrative review, we consider the psychotherapeutic applications of computer-based and online avatar technology; specifically, the ways in which avatars have been used to replace or augment traditional models of client–therapist interaction and communication. We additionally synthesize the functions that avatars can serve in such applications and consider the advantages and challenges of implementing this novel technology within e-mental health interventions.

## Applications of Avatars in e-Mental Health: An Overview

### Online Peer Support Communities

The potential to foster cohesive social networks is cited as a strength of virtual worlds in which users can interact with each other by adopting personalized avatars (1). Most attention has focused on the program Second Life, where users can create realistic-looking human avatars that they can use to manipulate stimuli within the virtual environment and remotely interact with other users – also represented as avatars – *via* text and/or audio.

In 2008, there were 68 health-related activities on Second Life, 20% of which were intended primarily for peer support (6). Many peer communities focused on sensitive topics (e.g., sexual health, addictions) or were organized for and by groups of people who are vulnerable to marginalization and discrimination in “real life” (e.g., people with disabilities). The popularity of Second Life peer support communities, and additional health-related activities (e.g., health promotion and education), may result from users’ ability to collaborate, interact, and consult with other avatars in real time while maintaining their anonymity (6). By 2013, only 24 health-related Second Life sites were still active, many of which were absent of other users when researchers entered the virtual host spaces (7). Thus, user anonymity may have actually minimized investment in building long-term relationships.

### Avatar Use to Replicate Traditional Psychotherapy Models in Online Virtual Environments

The Second Life platform has strong potential to replicate models of individual and group-based treatments, but conducted entirely online, with both client and therapist interacting with each other in a virtual environment. To date, this model has been trialed in two uncontrolled studies using individual (8) and group (9) formats.

Yuen et al. (8) conducted a manualized, acceptance-based behavioral treatment for adults with social anxiety disorder, delivered entirely *via* Second Life. Participants (*n* = 14) and therapists (*n* = 3) met in a private, secure virtual therapy room for a 1-h individual treatment session each week for 12 weeks. During role-playing exposure exercises, sessions occurred in other virtual spaces relevant to the exposure scenario (e.g., giving a presentation in a virtual conference room), with confederate therapists facilitating each exercise by adopting pre-made avatars with diverse physical characteristics (e.g., age, gender, ethnicity). Intention-to-treat analyses for a range of mood, psychosocial, and social anxiety measures indicated large posttreatment and 12-week follow-up effect sizes. Future research comparing the intervention to a control condition is required.

Also delivered in Second Life, Hoch et al. (9) developed an 8-week relaxation and mindfulness group, with sessions delivered twice-weekly to groups of up to 10 participants. The first session of each week involved teaching the participants specific relaxation strategies. In the second session, participants met in a virtual teaching space designed to look peaceful (e.g., a virtual forest), where they were asked to review their practice of these strategies. Overall, mental health symptoms measured by the Symptom Checklist 90 decreased from pretreatment to posttreatment, although changes were not statistically significant. Future research may need to use gold-standard measures of specific psychopathology symptoms, which may be more sensitive to changes. However, participants reported that they appreciated the convenience of being able to participate remotely in a virtual group program and also commented that the anonymity of participation made the intervention material more approachable.

### Avatar-Assisted Face-to-Face Therapies

Several studies have utilized various forms of avatar technology to facilitate or augment treatments that are delivered with the face-to-face support of a therapist. Two models of these avatar-assisted therapies have been implemented, which are as follows: (1) applications that require the client to “embody” or represent themselves as an avatar in order to participate in the therapy and (2) applications that do not require the client to embody an avatar, but rather, require the client to *interact with another avatar*, be it the therapist or an “other.”

#### Embodied Use of Avatars by the Client

Using Second Life, Kandalaft et al. (10) delivered a manualized social skills training program to eight young adults with high-functioning autism spectrum disorder. Ten sessions were completed over 5 weeks, during which the therapist physically sat alongside and coached the participant through virtual role-playing scenarios. During each session, the therapist – represented as an avatar – directed participants to various virtual spaces (e.g., cafes, parks, shops) where they met with a confederate clinician – also represented as an avatar – to practice social interactions in diverse role-playing situations (e.g., attending a job interview). Clinician-administered neurocognitive measures of verbal and non-verbal emotional recognition significantly improved from pre- to post-program, suggesting that the program may improve elements of social communication typically impaired in people with autism.

Using a different commercially available avatar platform (*ProReal*), van Rijn et al. (11) used avatar-mediated communication as a component of face-to-face group therapy in a prison setting. Unlike Second Life avatars, ProReal avatars appear as androgynous, featureless human forms, which users can manipulate in terms of color, size, and expressive gestures. ProReal avatars can also be given virtual props to facilitate symbolic emotional expression, such that participants could use the avatars to explore and communicate their emotions to other group members. Group therapy sessions were 90-min long, ran for 6 weeks, and were facilitated by a counselor. Distress ratings measured by the CORE-10 did reduce from pretreatment to posttreatment, although not statistically significantly. Qualitative feedback suggested that the avatars supported participants to express emotions that were difficult to communicate verbally, and to develop empathy for other group members.

#### Avatar-Mediated and Augmented Therapeutic Interactions

Avatar software can offer clients unique scope to address or confront their symptoms, within a safe and controlled environment, with the support of a therapist. Leff et al. (12) utilized this technology as part of a novel treatment for persecutory auditory hallucinations, with participants (*n* = 26) asked to create an animated avatar face of the entity that they believed was talking to them. In conjunction with voice transformation software to distort the therapist’s speech, the therapist used the avatar to role play the person’s auditory hallucination during exercises designed to support the person responding to their voice more adaptively. Highly promising results were reported in a pilot trial (12), with reductions in hallucination severity relative to wait-list controls, and some participants reporting a remission of their voices. These findings are currently being examined in a full sale randomized controlled trial (RCT) (13).

The value of avatar technology for learning and practice of new social skills has been recognized by numerous authors (5, 14–16). Both Rus-Calafell et al. (14) and Peyroux and Franck (15, 16) have used avatars to simulate social situations as part of social cognitive remediation programs for people with psychotic disorders. In the twice-weekly, 8-week *Soskitrain* program (14), participants (*n* = 12) practiced social skills with a variety of expressive avatar characters in different situational environments. Therapists could control the avatar’s behaviors according to participant responses to promote scaffolded learning and stop the interaction for therapeutic discussion. In an uncontrolled pilot trial of the program, Rus-Calafell et al. (14) reported significant pretreatment to posttreatment improvements to participants’ self-reported negative symptoms, social avoidance, and social functioning. System-recorded facial emotion recognition errors and time spent in avatar-based conversations also improved. Gains were maintained at 4-month follow-up. Using a different avatar-based simulation program (*RC2S*), Peyroux and Franck (16) reported two experimental single-case studies that made significant pretreatment to posttreatment improvements in theory of mind abilities and improved facial emotion recognition, social knowledge, self-esteem, and attributional style. Using RC2S (15, 16), participants learned to analyze the mental state, emotions, and intentions of “Tom,” an avatar character, to guide Tom’s responses to various social situations. As in Soskitrain, the therapist’s role was to provide social skills training and feedback to support participant’s interactions with the avatar.

### Avatars to Participate in “Serious Games”

Many video games require that the player embodies an avatar to interact with other players or to interact with automated non-player characters. In “serious games,” such game-like elements are incorporated into computerized psychotherapies to achieve a serious health-related goal (e.g., to reduce depression symptoms) (17). SPARX is an example of a serious game in which the participant, as an avatar, progresses through seven modules of a fantasy-based computerized game (18, 19), which incorporates cognitive–behavioral therapy strategies to treat depression in adolescents. At the commencement of each module, participants meet with an automated guide whose role is akin to a therapist. The guide informs participants of their tasks for each module and how they relate to improving depression. At the end of each module the guide provides a summary of what was learned. SPARX has shown efficacy in two RCTs, and participants have reported high levels of treatment satisfaction and acceptability (18, 20).

Unlike aforementioned avatar-based therapies (8–12), SPARX is an entirely self-guided e-mental health intervention. Participant feedback suggested that the self-guided nature of the program was one of its strengths, as were gaming elements that supported treatment engagement such as a story-like narrative throughout the seven modules, and automated characters that were perceived as being warm and caring (21).

### Avatars as Autonomous Virtual Therapists

Finally, avatars have been utilized as autonomous virtual therapists – also referred to as embodied communicative/relational agents – to facilitate the clinical interview and assessment process, to provide psychoeducation, or to direct individuals to access alternative psychological services (22–25). In these applications, the client is not required to embody an avatar to interact with the therapeutic agent, as in Second Life (8–10) and SPARX (18–20). Furthermore, the therapeutic agent, which can be represented as a realistic-looking human avatar (22–24) or as a two-dimensional animated character (25), is not controlled by a human clinician. Rather, the avatar is an autonomous agent presented on a computer monitor, which responds to the client’s text-based, auditory, and/or sensory input on the basis of artificial intelligence or algorithm.

To investigate user experiences, Rizzo et al. (22) conducted clinical interviews with 91 adults who interacted with an autonomous virtual therapist called “Ellie.” Their experiences were compared to 120 participants who interacted with a clinician-operated version of Ellie and 140 participants who participated in face-to-face clinical interviews. Most participants reported that they were willing and felt comfortable to share information with Ellie (in both autonomous and clinician-operated conditions), which is consistent with other studies that have found participants highly rate the therapeutic alliance with relational agents (25). Ratings of rapport and listening skills were significantly greater for the clinician-operated avatar than the autonomous avatar, and rapport ratings for the clinician-operated avatar exceeded those of face-to-face clinical interviewers. This may indicate the importance of realistic transactional elements in virtual interactions for reducing emotional barriers to client engagement (22); however, this suggestion requires investigation.

Pinto et al. (23, 24) investigated the efficacy of an avatar-based self-management intervention for young adults with depression (Electronic Self-Management Resource Training for Mental Health; eSMART-MH). *Via* laptops, participants accessed a virtual primary health clinic to communicate with simulated avatar health-care professionals. The program was designed to provide young people with training and practice in communicating with health-care professionals about depression, and to learn self-management strategies. An RCT (23) demonstrated that self-reported depression symptoms decreased significantly more for the eSMART-MH group (*n* = 12), when compared to an attentional control group; however, there was no significant change in symptoms from baseline to 12-week follow-up in either group. Participant feedback for future versions of the intervention included greater range of user input and avatar response options, the possibility to receive counseling, and access to the program *via* mobile devices (24).

## Therapeutic Functions and Challenges of Avatar Technology

### Supporting the Therapeutic Relationship through Virtual Presence

As the reviewed studies demonstrate, several applications have involved both the therapist and client utilizing avatars as a form of virtual embodiment (8–10), which allows both parties to feel a sense of social presence within a remotely accessed online environment. This sense of social presence, and the tendency for people to engage in greater self-disclosure during computer-mediated communication compared to face-to-face interactions (26), has strong potential to facilitate the development of online therapeutic relationships and may be more important for presentations where shame or stigma are central features. Indeed, feelings of copresence, emotional closeness, and interpersonal trust are equivalent for avatar-, audio-, and video-based modes of communication (27). In line with the contention that avatars can generate social presence in virtual environments, participants in a number of the studies listed above noted that their interactions fostered a sense of genuine rapport (8, 10), even when the real/implied therapist was operated on the basis of automated therapeutic scripts (21, 24) or artificial intelligence (22).

### Reduction of Communication Barriers

The flexibility to use audio- and/or text-based communication in avatar platforms provides options for clients to choose a communication mode with which they feel most comfortable. For instance, Stendal and Balandin (28) illustrated how text-based communication in Second Life reduced communication barriers for a participant with autism spectrum disorder by reducing the ambiguity of social and emotional cues during his interactions with online peers. The participant reported feeling a sense of security and control that he perceived his disability did not afford him in the “real world,” empowering him to develop personally valued online friendships. Relatedly, participants with autism spectrum disorder in Kandalaft et al.’s study (10) suggested that being comfortable with computer-mediated communication supported their confidence to participate in the avatar-simulated social situations. For many individuals with physical, mental, and language impairments, the ability to communicate *via* an online medium of choice and from the perceived safety of one’s home is likely to be an advantage of avatar-based technology.

Like other forms of online communication, however, technical difficulties can create new communication barriers that impact on the quality of treatment sessions (e.g., flow, timing) (8), and clients must feel confident to use (or learn to use) the technology in the first instance. Another issue to consider is that the lack of visual cues may reduce a sense of accountability to one’s conversational partner, as both parties can engage in other tasks without the other being able to see this occurring. Even for therapists, this can foster a sense of disconnectedness, which itself can inhibit the development of communicational and emotional attunement (29).

### Anonymity to Promote Treatment-Seeking

Unlike videoconferencing, avatars afford the possibility of anonymous engagement with psychological services. Potentially, this may attract individuals to receive treatment they may otherwise not seek (e.g., due to shame or stigma) by engaging them wholly in an avatar-based e-mental health intervention (29) or by encouraging them to seek face-to-face treatment following an anonymous and positive interaction with an autonomous virtual therapist (22, 24). A case study by Quackenbush and Krasner (29) highlighted how a client who was reluctant to engage in online treatment *via* videoconferencing due to fear of racial discrimination was successfully treated in Second Life, where the client was able to use an avatar and pseudonym that disguised his ethnicity.

Reflecting ethical concerns relating to risk management and client safety, anonymous use of avatar technology has been recommended only for instances in which the client is seeking generic psychoeducation and referral information (22, 30). When ongoing avatar-based psychotherapy services are offered, authentication of the client’s identity is advised (30). This can still permit pseudonymity and customization of one’s avatar. The psychological impact of a client’s “anonymized” avatar on both the client and therapist requires investigation; particularly in light of evidence that altering the visual features of an avatar (e.g., height, attractiveness) can both positively and negatively affect the individual’s virtual and “real world” behavior (31).

### Exploration of Client Identity

A particularly novel component of (embodied) avatar use in online psychotherapeutic interventions is the capacity they provide for clients to express, experiment with, explore, and construct a virtual, visual representation of their identity. In the context of using Second Life for social purposes, three functions of avatars as an expression of the individual’s “real world” identity have been identified (32). First, avatars can be utilized as a vehicle for engaging in a virtual world, whereby the individual’s representation of their true physical characteristics, and their real name, occupation, interests, etc., were conveyed *via* their avatar. Second, avatars could be utilized to enhance one’s real world self by embodying features perceived by the individual as positive; for instance, customizing the avatar to appear more youthful or expressing personality traits the individual normally suppresses. Finally, avatars could be utilized to diversify one’s identity by adopting an entirely new identity in the virtual world; for instance, adopting a new name, different gender, creating a new “life story,” and visually representing one’s avatar in a way that does not reflect one’s true physical appearance. Participants reported that regardless of whether they utilized their avatar for self-extension, enhancement, or diversification, experiences within Second Life generalized to positive physical, cognitive, social, or emotional outcomes in the real world. Such identity-based functions could have significant implications for the therapeutic use of avatars.

### Manipulation and Control of Treatment Stimuli

Finally, the ability for the therapist to control the content of virtual stimuli and intensity of virtual situations as part of behavioral interventions is a key advantage of avatar technology over other forms of computer-mediated communication (1). As with virtual reality exposure therapy conducted through head-mounted display devices, computer-based virtual reality applications can be conducted remotely (8, 9) or within the therapist’s office (10–16), thereby offering a safe environment for exposure-based treatments; compared to *in vivo* therapies, sessions utilizing the same exposure stimuli or role-playing scenarios can feasibly and conveniently be repeated as often as needed for the client to learn new skills; and clients may feel a greater sense of control over and safety within virtual settings, which could support treatment adherence. Additionally, computer-based applications are unlikely to induce the nausea and sensory distortions that can be experienced with virtual reality technology (33).

Research is required to determine whether computer-based virtual reality applications can generate an equivalent sense of realistic “presence” as do head-mounted simulation technologies; the assumption being that presence will elicit the level of anxiety required for habituation over the course of exposure therapy (34). The role of presence as a mechanism of behavior change requires greater investigation across all modalities of virtual therapies, as does the level of photorealism required of avatars and virtual environments to induce a sense of being immersed in a therapeutic interaction. This may depend on the needs of the clinical population undergoing treatment. For instance, the high drop-out rate (34.6%) from Leff et al.’s (12) treatment for auditory hallucinations may have indicated that use of avatars to represent experiences more concretely may have been too confronting for many participants to tolerate.

## Conclusion

The use of avatars in e-mental health interventions represents a nascent area of inquiry. As demonstrated in this review, the psychotherapeutic applications of computer-based and online avatar technologies have been diverse. Several of the studies reviewed in this paper were uncontrolled trials with small sample sizes and framed as pilot investigations of feasibility as opposed to efficacy. The diversity in study aims, methodologies, participant groups, intervention types, outcome measures, technologies, and treatment delivery models prevents a conclusion as to the efficacy of avatars in delivering e-mental health interventions – nor was this the aim of this review. However, this diversity does highlight its significant potential and functionality.

As a flexible and creative platform with which to deliver individual and group therapies, peer support, and as a form of e-mental health augmentative intervention, avatar technology offers significant potential to engage a broad range of clients in need of psychological support who may otherwise be unable or unwilling to participate in traditional treatment models. In particular, avatars may foster the development of a strong virtual therapeutic alliance; overcome communication barriers experienced by individuals with various disabilities and mental disorders; offer an anonymized means of seeking treatment; support clients to explore and extend their identity; and provide therapists with greater control over treatment stimuli that involve an element of exposure or skills training. Nevertheless, there appear to be many challenges to the ethical and feasible implementation of avatar-based e-mental health interventions. In light of advancing portable technologies, such as smart phones and tablet devices, it will be interesting to observe how the models of avatar-based e-mental health interventions will further diversify and enable greater access to engaging interventions. Delineating feasible and appropriate models of avatar use for psychotherapeutic purposes, and investigating both consumer and clinician attitudes and preferences toward the technology for this purpose, will be an important endeavor for future research.

## Author Contributions

EF conducted the literature review, and IR drafted the first version of the manuscript. NT, KW, JA, and MK contributed to and edited the final manuscript.

## Conflict of Interest Statement

The authors declare that the manuscript was developed in the absence of any commercial or financial relationships that could be construed as a potential conflict of interest.
